# A Novel Lipid Prognostic Signature of ADCY2, LIPE, and OLR1 in Head and Neck Squamous Cell Carcinoma

**DOI:** 10.3389/fonc.2021.735993

**Published:** 2021-11-25

**Authors:** Xiaolei Gao, Na Zhao, Liying Dong, Xuan Zheng, Yixin Zhang, Chong Ding, Shuyan Zhao, Zeyun Ma, Yixiang Wang

**Affiliations:** ^1^ Central Laboratory, Peking University School and Hospital of Stomatology, Beijing, China; ^2^ Department of Oral and Maxillofacial Surgery, Peking University School and Hospital of Stomatology, Beijing, China; ^3^ National Engineering Laboratory for Digital and Material Technology of Stomatology, Peking University School and Hospital of Stomatology, Beijing, China; ^4^ Beijing Key Laboratory of Digital Stomatology, Peking University School and Hospital of Stomatology, Beijing, China; ^5^ Department of Restorative Dentistry and Biomaterials Sciences, Harvard School of Dental Medicine, Boston, MA, United States; ^6^ Department of Prosthodontics, Shanghai Stomatological Hospital, Fudan University, Shanghai, China; ^7^ Shanghai Key Laboratory of Craniomaxillofacial Development and Diseases, Shanghai Stomatological Hospital, Fudan University, Shanghai, China; ^8^ The Fifth Clinical Division, Peking University School and Hospital of Stomatology, Beijing, China; ^9^ Department of VIP Service, Peking University School and Hospital of Stomatology, Beijing, China

**Keywords:** head and neck squamous cell carcinoma, survival, lipid-related prognostic signature, TP53 status, immune characteristics

## Abstract

**Simple Summary:**

Clinically, aberrant lipid metabolism is responsible for overweight and/or obesity. Overweight is considered as an independent factor of cancer risk in 2019. Therefore, lipid metabolic reprogramming is an emerging hallmark of malignancy. It is an urgent need to comprehensively understand the relationship among lipid metabolism and HNSCC and identify a valuable biomarker for predicting prognosis of HNSCC patients. Three new findings were found in this study. Firstly, we identified the lipid-related differentially expressed genes (DEGs) by using the GEO microarrays and TCGA dataset. A novel lipid-related mRNA prognostic signature (LRPS, consisting of ADCY2, LIPE and OLR1) was developed, which could predict the survival and prognosis of HNSCC patients as an independent effective prognostic factor. Secondly, we found that the LRPS could indicate the type of infiltrated immune cells in HNSCC tumor microenvironment. Thirdly, we verified that the LPPS score could interpret the TP53 status of HNSCC. Our new findings indicated that LRPS has a potential to be a promising indicator of overall survival, TP53 status, and immune characteristics in HNSCC, and perhaps can monitor and guide the treatment efficacy and prognosis of HNSCC in the future.

**Background:**

Head and neck squamous cell carcinoma (HNSCC) is characterized by a high frequency of lymph node metastasis and a high mortality. Lipid metabolic reprogramming is an emerging carcinogen as its role in fulfilling cancer growth and spread. However, little is known about the correlation between lipid metabolism and HNSCC.

**Materials and Methods:**

Expressions of lipid-related genes were obtained from the Cancer Genome Atlas (TCGA) and Gene expression Omnibus (GEO) databases for differential and functional analyses. A total number of 498 patients from TCGA with complete information were included to identify a lipid-related prognostic signature (LRPS), based on ADCY2, LIPE, and OLR1, by using univariate and multivariate Cox regression analyses. LRPS-high and LRPS-low groups were accordingly divided to pathway and cell enrichment analyses.

**Results:**

LRS-low patients had a better overall survival and relapse - free survival than LRS-high ones in HNSCC. The LRPS-high group was significantly related to perineural invasion of cancer, cancer-related pathways, high TP53 mutation rate, high proportion of natural killer T cells (NKT), dendritic cells, monocytes, Treg, and M1 and M2 macrophage infiltration in HNSCC tumor tissues. Conversely, the LRPS-low group correlated with DNA damage-related and T-cell-regulated pathways, low frequency of mutated TP53, and high infiltration of B cells and CD4+ effector cells including Th1 and Th2.

**Conclusion:**

LRPS has a potential to be a promising indicator of overall survival, prognosis, TP53 status, and immune characteristics in HNSCC.

## Introduction

Head and neck squamous cell carcinoma (HNSCC) is the most common type of the head and neck cancers, with a high risk for recurrence and poor survival under the advanced treatment approaches. The incidence of HNSCC was increased by 36.3% during the past 10 years, from ~482,000 HNSCC patients in 2008 to ~657,000 cases in 2018 (1, 2). Smoking, alcohol assumption, and virus infection are recognized as important carcinogenic factors (3). Recent studies implicate that abnormal lipid metabolism may be related with HNSCC development and progression (4, 5).

Lipid metabolic reprogramming is an emerging hallmark of malignancy (6). Overwhelming lipid anabolic and catabolic processes are essential for the uncontrolled cell proliferation and rapid cancer growth. Simultaneously, lipids constitute most of the cell membranes and serve as signaling molecules. Theoretically, fatty acids and cholesterol synthesis provide carcinogenesis and metastasis with a range of metabolic fuels and substrates, as well as pro-tumor signaling cytokines (7–11). Furthermore, the roles of lipid metabolites in protecting cancer cells from harmful conditions (like endoplasmic reticulum stress, reactive oxygen species, and drug toxicity) have been substantiated in various cancers (12, 13). Some oncolipid-activated signaling pathways, such as sterol regulatory element-binding proteins and stearoyl-CoA desaturases, have been identified to be the potential targets for cancer treatment in the future (6, 14, 15).

Clinically, aberrant lipid metabolism is responsible for overweight and obesity. Overweight is considered as an independent factor of cancer risk by the American Cancer Society, which released a report entitled Cancer Facts & Figures in 2019 (16). Nowadays, it is estimated that 5% of cancers in men and 11% in women are attributed to overweight (17). Experimental evidence indicates that high-fat diet-induced obesity not only promotes carcinogenesis, but also induces lymphangiogenesis and lymphatic metastasis *in vivo* (18–20). Conversely, diet-caused weight loss was shown to reduce cancer risk (21). Furthermore, a deliberate weight loss has been proved to reverse the effects of obesity-induced oxidative stress, inflammatory activities, and oncogenesis (22). Reduction of DNA damage responses in overweight mice was also observed after an administration of energy restriction (23).

In this study, we firstly identified the lipid-related differentially expressed genes (DEGs) by using the GEO microarrays and the TCGA dataset. A novel lipid-related mRNA prognostic signature (LRPS, consisting of ADCY2, LIPE, and OLR1) was developed for predicting survival of HNSCC patients. Accordingly, HNSCC patients were divided into high-risk and low-risk groups according to their LRPS signature, and gene-set enrichment analysis (GSEA) and cell enrichment analysis were used to elucidate the potential mechanisms.

## Materials and Methods

### Ethics Approval

The original datasets in our study were downloaded from the TCGA database and GEO dataset. We downloaded and analyzed the study data in accordance with the relevant data policies of TCGA database and GEO datasets, and therefore, no additional ethics approval was needed.

### Data Source

The original datasets comparing the mRNA expression profiles between tumors and adjacent normal tissues were obtained from the three GEO databases [GSE30784 (containing 167 oral squamous cell carcinoma, 17 dysplasia, and 45 normal oral tissues), GSE37991 (containing 40 male oral squamous cell carcinoma biopsies), and GSE65858 (containing 290 HNSCC biopsies)] and a TCGA dataset (containing 498 HNSCC biopsies). The clinical samples from the TCGA database with complete clinical information of patients were selected. The microarray data of GSE30784, GSE37991, and GSE65858 were based on GPL570 (Affymetrix Human Genome U133 Plus 2.0 Array), GPL6883 (Illumina HumanRef-8 v3.0 expression beadchip), and GPL10558 (Illumina HumanHT-12 V4.0 expression beadchip), respectively. The corresponding clinical information of patients with HNSCC was also acquired from the TCGA database (up to July 19, 2019). A total of 498 HNSCC patients with detailed follow-up time were included for the following analyses.

### Data Processing and Differential Expression Analysis

The GEO data were processed and analyzed using GEO2R (https://www.ncbi.nlm.nih.gov/geo/geo2r/). TCGA mRNA counts were normalized and analyzed by R packages (DESeq2 package) (*p* < 0.01, |log2FC| > 2).

### Functional Enrichment Analyses

Gene ontology (GO) and KEGG pathway enrichment analyses were performed using the DAVID online database (the Database for Annotation, Visualization, and Integration Discovery) (24, 25). The enriched biological processes (BP), cellular component (CC), and molecular function (MF) were obtained to analyze the DEGs.

### Protein–Protein Interaction Analysis

The STRING online database (http://string-db.org) was performed for PPI analysis. Cytoscape software was employed to construct the PPI network (26). MCODE tool of Cytoscape was performed to identify gene cluster of the PPI network. Degree cutoff ≥ 2, node score cutoff ≥ 0.2, K-core ≥ 2, and max depth = 100 was set as the threshold value.

### Prognostic Signature Generation and Validation

The TCGA original dataset was performed as a training cohort. Univariate and multivariate Cox proportional hazards regression analyses were carried out to identify potential genetic predictors for HNSCC survival. Kaplan–Meier survival analysis with log-rank test was performed in R package. An internal dataset derived from the original TCGA served as a validation cohort using the bootstrap resampling method (27). Multivariate survival analysis was then performed to assess the association between the signature and clinical pathological index, namely, age, gender, lymphovascular invasion, margin status, recurrence, lymphatic metastasis, perineural invasion, cancer status, and nodal extracapsular spread.

### Pathway Enrichment and Immune Enrichment Analyses

Gene-set enrichment analysis (GSEA) was performed using GSEA software with the criteria *p* < 0.05 and FDR < 0.25 (28, 29) and visualized using clusterProfiler packages of R (30). mRNA expression profiles were uploaded to xCell online software to evaluate the immunocyte heterogeneity of LRPS-high and LRPS-low groups (31).

### Statistical Analysis

Statistical analyses were performed using GraphPad Prism 8.4 and R software (version 3.6.3); *p* < 0.05 was considered statistically significant. A nonparametric *t-*test was performed to compare continuous variables and *χ* ^2^ test was used to compare categorical variables between two groups. ANOVA test was utilized to compare more than two groups.

## Results

### Lipid-Associated DEGs in HNSCC

**Figure 1:**
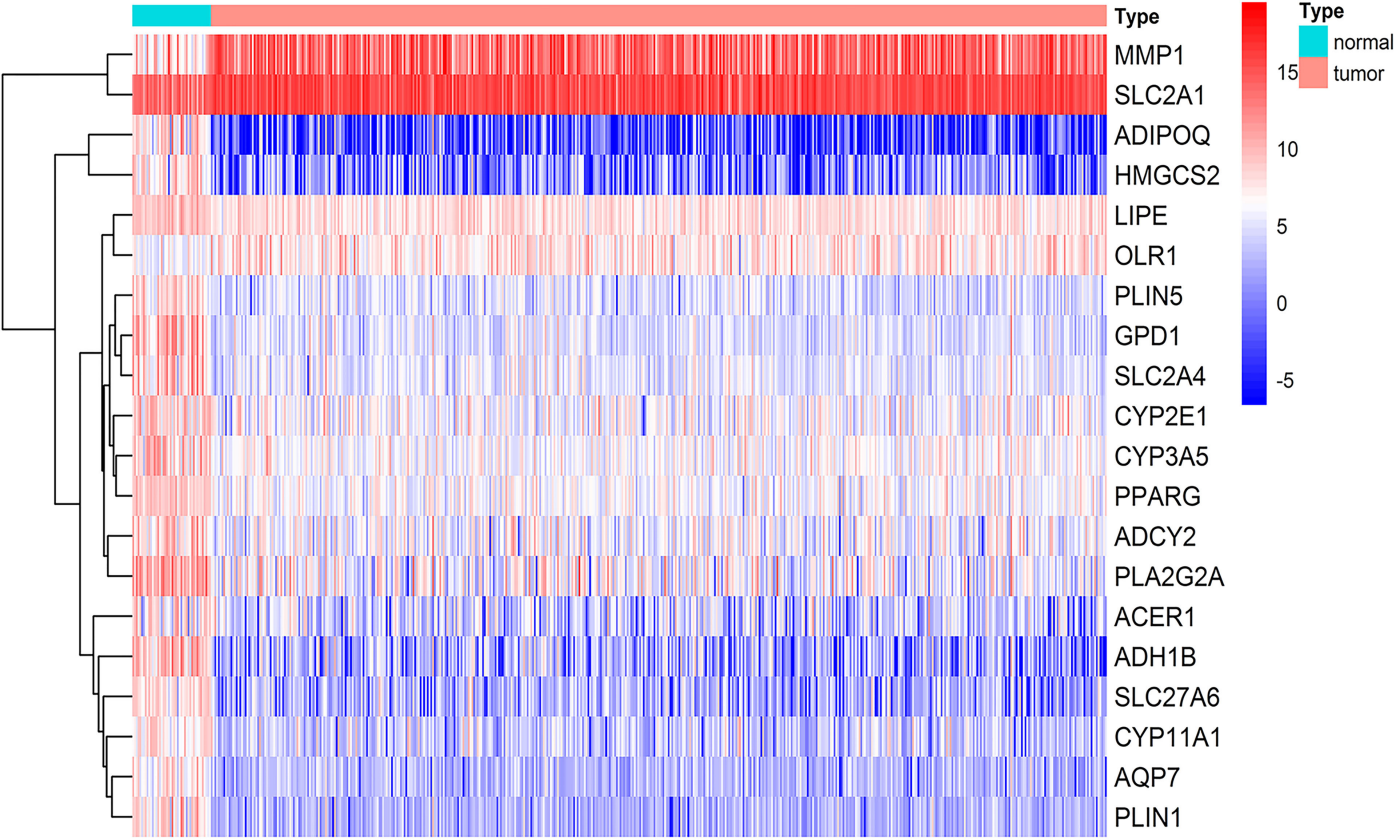
Lipid-related differentially expressed genes (DEGs) in HNSCC. Top 20 of the 65 genes involving lipid metabolism showed abnormal expression in HNSCC from the TCGA database including 44 normal tissues and 502 tumors (*p* < 0.01, |logFC| > 2). The color from blue to red represents the gene expressions from high to low between tumors *vs.* normal tissues.

To explore the lipid-related genes in HNSCC, 37 lipid-metabolic channels and 4 lipid-related signaling pathways (**Supplementary Files S1**) were selected based on KEGG pathway databases, and then analyzed in the TCGA database and GEO datasets using R packages. The result showed that a total of 65 genes significantly abnormally expressed in all three independent cohorts including TCGA, GSE30784, and GSE37991. The 26 upregulated and 39 downregulated lipid-related DEGs in total are listed in **Table 1**. The top 20 DEGs from the TCGA database are listed in **Figure 1**, and all lipid-related DEGs in GEO datasets are shown in **Supplementary Figure S1** (*p* < 0.01, |log2FC| > 2).

**Table 1:** The lipid-related DEGs among GSE30784, GSE37991, and TCGA.

Genes	LogFC	*p*-value	Functions
APOC2	2.17	5.02E−08	Cholesterol metabolism
ADCY3	0.71	1.08E−10	Regulation of lipolysis in adipocytes
ACOT7	1.23	3.22E−19	Fatty acid elongation
CYP27B1	2.48	4.06E−20	Steroid biosynthesis
CERS2	0.62	1.42E−10	Sphingolipid metabolism
DHCR7	1.01	3.61E−06	Steroid biosynthesis
GLA	0.76	1.09E−12	Sphingolipid metabolism; glycerolipid metabolism
GPX7	1.00	8.32E−07	Arachidonic acid metabolism
GPX8	1.28	1.69E−14	Arachidonic acid metabolism
GNAI1	0.76	7.48E−09	Regulation of lipolysis in adipocytes
HSD17B6	2.11	3.21E−37	Steroid hormone biosynthesis
HACD3	0.66	4.74E−13	Fatty acid elongation; fatty acid metabolism; biosynthesis of unsaturated fatty acids
LPCAT1	1.72	8.18E−27	Glycerophospholipid metabolism; ether lipid metabolism
MMP1	3.08	1.22E−13	PPAR signaling pathway
OLR1	2.04	7.42E−10	PPAR signaling pathway
PCSK9	2.77	1.62E−19	Cholesterol metabolism
PLPP4	4.34	3.17E−27	Glycerophospholipid metabolism; glycerolipid metabolism
PIK3CD	1.24	3.39E−16	Regulation of lipolysis in adipocytes
PPT1	0.77	1.85E−15	Fatty acid elongation; fatty acid metabolism
PLA2G7	1.98	2.09E−17	Ether lipid metabolism
SCARB1	0.73	3.19E−06	Fat digestion and absorption; cholesterol metabolism
SLC16A1	1.37	2.64E−17	Fatty acid biosynthesis
SQLE	0.82	4.82E−08	Steroid biosynthesis
SCD5	0.93	6.99E−08	Fatty acid metabolism; biosynthesis of unsaturated fatty acids; PPAR signaling pathway; AMPK signaling pathway
SPHK1	1.08	1.68E−11	Sphingosine degradation; sphingolipid metabolism
SLC2A1	1.76	1.40E−18	Adipocytokine signaling pathway
ADH1B	−5.36	9.21E−79	Fatty acid degradation
ADCY2	−2.20	1.24E−16	Regulation of lipolysis in adipocytes
AQP7	−4.11	2.31E−94	Regulation of lipolysis in adipocytes
ALOX12	−2.51	1.43E−29	Arachidonic acid metabolism
ACER1	−2.50	8.28E−17	Sphingosine biosynthesis; sphingolipid metabolism
ADIPOQ	−5.67	7.00E−43	Adipocytokine signaling pathway; PPAR signaling pathway; AMPK signaling pathway
ASPG	−1.47	2.44E−10	Cholesterol metabolism
ACADSB	−1.68	1.66E−43	Fatty acid degradation; fatty acid metabolism
ADH7	−1.79	8.69E−09	Fatty acid degradation
CYP3A5	−3.05	3.18E−54	Steroid hormone biosynthesis
CYP11A1	−2.21	4.01E−15	Steroid hormone biosynthesis
CAB39L	−2.14	6.29E−111	AMPK signaling pathway
CHPT1	−1.64	5.19E−23	Ether lipid metabolism; glycerophospholipid metabolism; phosphatidylcholine (PC) biosynthesis
CH25H	−1.60	1.70E−16	Primary bile acid biosynthesis
CYP2E1	−1.89	9.28E−14	Steroid hormone biosynthesis; arachidonic acid metabolism; linoleic acid metabolism
CYP2J2	−1.63	1.15E−25	Arachidonic acid metabolism; linoleic acid metabolism
DEGS2	−1.69	5.96E−14	Ceramide biosynthesis; sphingosine biosynthesis; sphingolipid metabolism
EPHX2	−1.63	3.40E−26	Arachidonic acid metabolism
FABP3	−3.03	3.73E−49	PPAR signaling pathway
GDPD3	−2.25	4.37E−30	Ether lipid metabolism
GPX3	−2.45	2.91E−44	Arachidonic acid metabolism
GPD1L	−2.66	2.88E−117	Glycerophospholipid metabolism
GPD1	−5.09	6.26E−148	Glycerophospholipid metabolism
HMGCS2	−4.71	5.67E−51	Synthesis and degradation of ketone bodies; PPAR signaling pathway; MVA pathway
LIPE	−1.35	7.95E−18	Regulation of lipolysis in adipocytes
MGLL	−1.95	1.49E−44	Regulation of lipolysis in adipocytes; glycerolipid metabolism; acylglycerol degradation
PLA2G2A	−2.52	8.11E−15	Fat digestion and absorption; ether lipid metabolism; glycerophospholipid metabolism; arachidonic acid metabolism; linoleic acid metabolism; alpha-linolenic acid metabolism
PLIN1	−5.44	4.74E−149	Regulation of lipolysis in adipocytes; PPAR signaling pathway
PLIN4	−4.40	6.43E−114	PPAR signaling pathway
PLIN5	−3.28	5.54E−69	PPAR signaling pathway
PLA2G16	−1.48	2.10E−13	Regulation of lipolysis in adipocytes; ether lipid metabolism; glycerophospholipid metabolism; arachidonic acid metabolism; linoleic acid metabolism; alpha-linolenic acid metabolism
PTGDS	−1.76	2.86E−16	Arachidonic acid metabolism
PPARG	−2.21	5.74E−29	PPAR signaling pathway
SLC2A4	−4.30	6.92E−102	Adipocytokine signaling pathway
SLC27A6	−3.55	1.00E−38	PPAR signaling pathway
SORBS1	−2.76	3.87E−64	PPAR signaling pathway
SULT2B1	−1.50	1.13E−11	Steroid hormone biosynthesis
SORT1	−1.43	3.82E−31	Cholesterol metabolism
TM7SF2	−1.82	5.62E−26	Cholesterol biosynthesis; steroid biosynthesis

LogFC, log Fold Change.

### Comprehensive Analysis of Molecular Characteristics in DEGs

**Figure 2:**
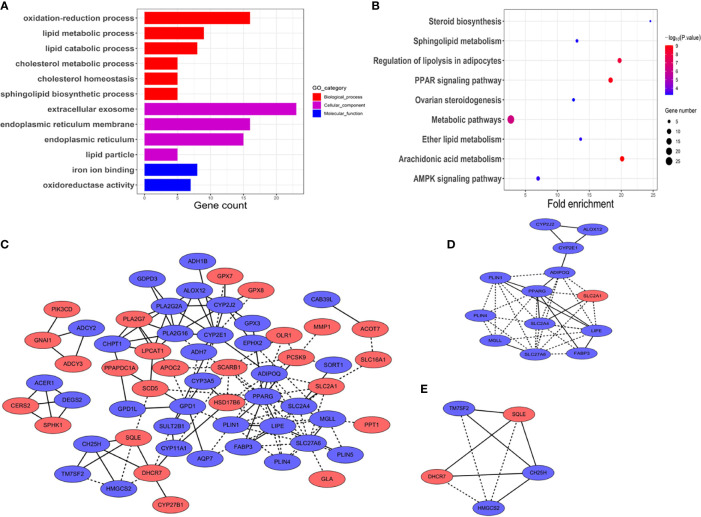
Functional vanalyses and PPT network of the DEGs. **(A)** GO analysis including biological processes, cellular components, and molecular functions. The *x*-axis shows gene counts enriched in these processes. (*p* < 0.01, FDR < 0.05). **(B)** Pathway analysis of the DEGs showed the common pathways in the KEGG database. The size of the points represented the numbers of the enriched genes; the bigger the size, the more genes enriched. Blue to red points represented statistical significance from low significance to high significance (*p* < 0.01, FDR < 0.05). **(C)** PPI network of the DEGs. Blue represents down-expressed genes, and red denotes up-expressed genes. Solid lines represent known interactions from curated databases or experimentally determined. Dotted lines represent predicted interactions. **(D, E)** Key module genes, namely, module 1 and module 2, with scores of 6.17 and 4.5, respectively.

Potential functions were then investigated in biological processes of the DEGs in HNSCC. GO analysis was performed and visualized in **Figure 2A**. The module of BP showed that the oxidation–reduction process, cholesterol homeostasis, sphingolipid biosynthetic process, and lipid metabolic and catabolic processes were commonly enriched. CC showed that the DEGs were significantly enriched in extracellular exosome, endoplasmic reticulum (membrane), and lipid particle. With regard to the module of molecular function (MF), the DEGs were mainly involved in iron ion binding and oxidoreductase activity.

Next, KEGG pathway enrichment analysis was used to figure out functions of the proteins encoded by the DEGs. As shown in **Figure 2B**, the DEGs were closely associated with arachidonic acid metabolism, PPAR signaling pathway, regulation of lipolysis in adipocytes, and metabolic pathways. *p* < 10^−5^ was recognized as significantly enrichment categories.

To figure out the relationship between the DEGs in HNSCC, the PPI network was constructed by STRING online database (**Figure 2C**). The central node genes (more than 10 connections or interactions) and the top 10 highly connected genes were identified, namely, PPARG, LIPE, SLC27A6, CYP2E1, ADIPOQ, PLA2G16, PLIN1, PLA2G2A, CYP2J2, and SLC2A4 (**Supplementary Files S2**). MCODE plugin from Cytoscape was used for the key module within the PPI network. The two most significant modules were identified for further pathway enrichment analysis. Module 1 consisted of 13 hub genes, namely, LIPE, PPARG, ADIPOQ, SLC2A1, SLC2A4, SLC27A6, MGLL, PLIN1, PLIN4, FABP3, CYP2E1, ALOX12, and CYP2J2 (**Figure 2D**). Module 2 included 5 hub genes, namely, SQLE, DHCR7, HMGCS2, TM7SF2, and CH25H (**Figure 2E**). Pathway enrichment analysis revealed that the hub genes in module 1 were closely correlated with PPAR signaling pathway (*p* = 2.58 × 10^−6^) and AMPK signaling pathway (*p* = 8.29 × 10^−4^). Module 2 was mainly enriched in steroid biosynthesis (*p* = 4.80 × 10^−5^) (**Supplementary Files S2**).

### Identification of a Lipid-Related Prognostic Signature of HNSCC

**Figure 3:**
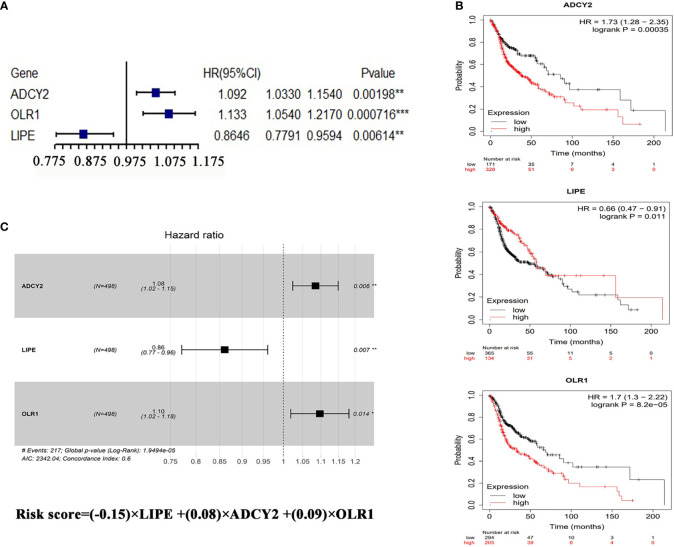
Prognostic analysis of LRPS genes. **(A)** Univariate Cox analysis of OLR1, ADCY2, and LIPE. **(B)** Survival analysis of the three genes in HNSCC according to the Kaplan–Meier Plotter online database (http://kmplot.com/analysis/index.php?p=background). **(C)** The risk score performed using multivariate Cox analysis of the three genes. *p < 0.05, **p < 0.01, ***p < 0.001.

To verify whether the lipid-related DEGs could be potential prognostic markers for HNSCC, the univariate and multivariate Cox analyses were performed to analyze the lipid-related DEGs as predictors for survival in TCGA patients with HNSCC (**Supplementary Files S3**, Model dataset). Univariate Cox analysis showed that ADCY2, OLR1, and LIPE significantly affected the overall survival of patients with HNSCC among the DEGs (**Figures 3A, B** and **Supplementary Files S4**). Next, a lipid-related prognostic signature (LRPS), containing LIPE, ADCY2, and OLR1, was constructed based on the coefficient of multivariate Cox analysis and mRNA expression of the three genes, the risk score = (−0.15) × LIPE + (0.08) × ADCY2 + (0.09) × OLR1 (**Figure 3C**). Subsequently, the LRPS containing LIPE, ADCY2, and OLR1 was selected to predict the prognosis of HNSCC patients through the TCGA and GEO databases.

**Figure 4:**
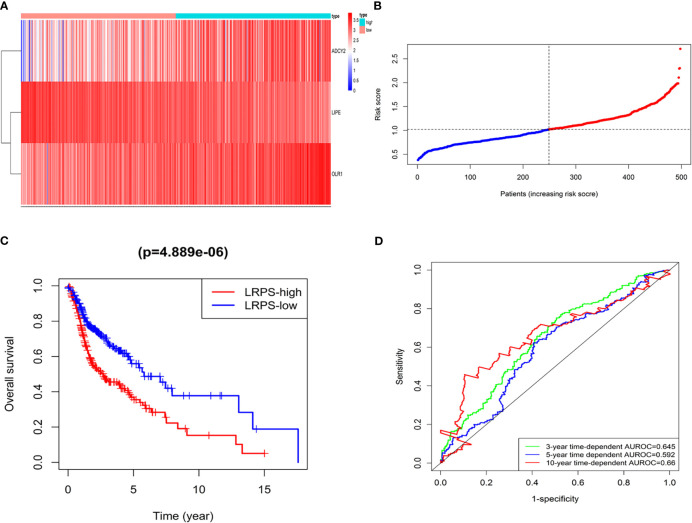
LRPS in the TCGA model dataset. **(A)** Heatmap for the mRNA expression distribution in TCGA cohort by risk score, with red representing high expression and blue representing low expression. **(B)** The risk scores for patients with HNSCC were plotted in ascending order. **(C)** The survival rates of HNSCC patients between the LRPS-high group and LRPS-low group (*p* = 4.889 × 10^−6^). **(D)** C-index values of ROC analysis.

The patients were accordingly divided into the high-risk group (*n* = 249) and low-risk group (*n* = 249) based on the risk score (**Figures 4A, B**), finding that the HNSCC patients in the high-risk group had a poorer 5-year overall survival (36.9%, HR = 0.377, 95% CI = 29.8%-45.7%) than those in the low-risk group (55.9%, HR = 0.566, 95% CI = 47.07%–66.5%) (*p* = 4.889 × 10^−6^) (**Figure 4C** and **Supplementary Files S5**). The concordance indices (C-index) of the lipid signature showed a higher specificity and sensitivity for predicting 3-, 5-, and 10-year overall survival (C-index = 0.645, 0.592, and 0.66, respectively, **Figure 4D**).

The LRPS was validated in the GSE65858 database. A total of 290 patients with HNSCC were subdivided into LRPS-high and -low groups according to the risk score, and survival analysis showed that the LRPS-high group had poorer 5-year overall survival than the LRPS-low group with a high effectivity (**Figures S2A, B**). We also established an internal TCGA dataset by bootstrap resampling method to validate the effectiveness of the LRPS (**Supplementary Files S3**, Validation dataset). The clinical characteristics within the two datasets had no significant differences using *t*-test analysis (**Supplementary Table S1**). The validation database was calculated and divided in the same way as the original group (**Figure S3**). Five-year overall survival analysis demonstrated that the high-risk group (37.92%, 95% CI: 30.71%–46.8%) was significantly poorer than its counterpart (59%, 95% CI: 51.1%–68.2%, *p* < 0.001, **Supplementary Files S5**). Taken together, the lipid-based signature of ADCY2, LIPE, and OLR1 could effectively predict the HNSCC patients’ survival.

### LRPS Was an Independent Indicator of Prognosis and Closely Correlated to HNSCC Recurrence

**Figure 5:**
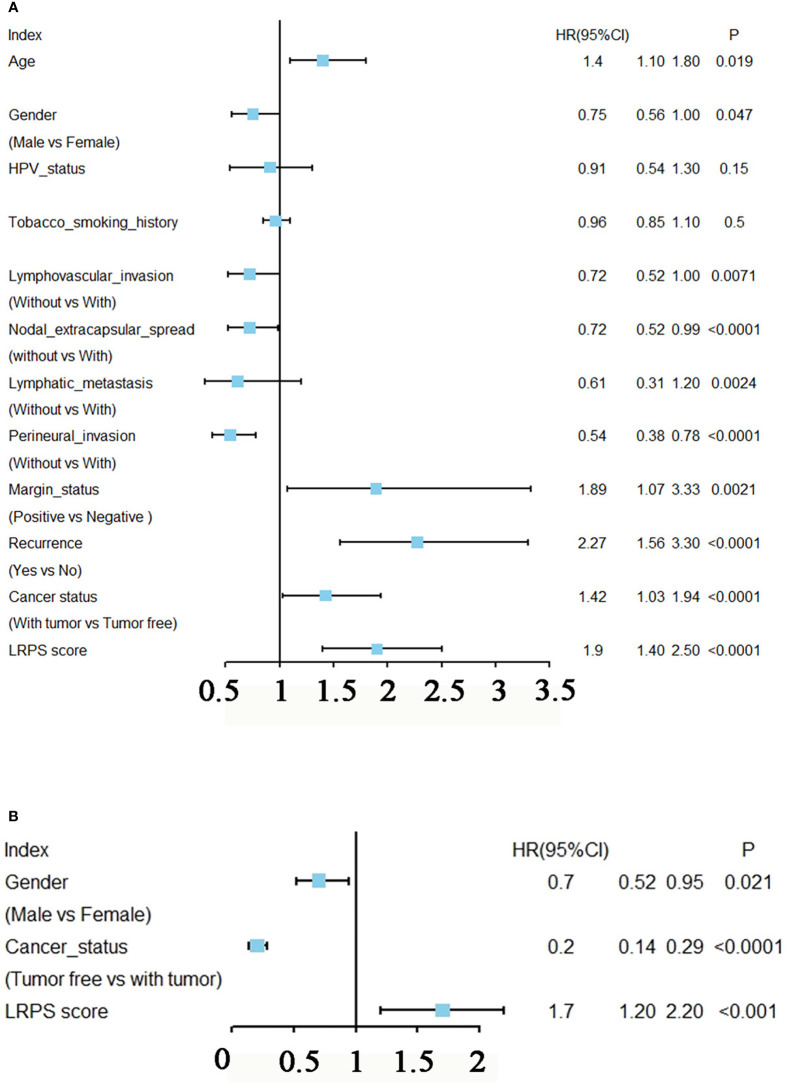
Prognostic analysis of LRPS in HNSCC. **(A)** Univariate Cox analysis of clinicopathologic factors and the LPRS score in TCGA-HNSCC patients. **(B)** Multivariate Cox analysis of the significant factors according to results from the univariate Cox analysis (*p* < 0.05).

Univariate Cox regression analysis showed that age, gender, lymphovascular invasion and metastasis, nodal extracapsular spread, perineural invasion, margin status, recurrence, cancer status, and LRPS score were significantly correlated with HNSCC prognosis (**Figure 5A**). Multivariate Cox analysis determined LRPS as an independent predictor after adjustment by other pathologic characteristics (**Figure 5B**). We evaluated the clinicopathologic factors in HNSCC among LRPS-high and LRPS-low groups (**Table 2**). Meanwhile, the LRPS could also independently predict the overall survival of HNSCC patients from the GEO database (**Figures S2C, D**). Statistically, LRPS-high patients were more prone to suffering relapse than the LRPS-low counterparts (52.73% *vs.* 22.92%, *p* = 0.0024). A high LRPS score had affinity relation with perineural invasion, compared with a low LRPS score (54.59% and 38.41% respectively, *p* = 0.0027). There were no significant differences within age, sex, alcohol and smoking history, tumor size and stage, lymphovascular invasion, and metastasis between the LRPS-high and LRPS-low groups.

**Table 2:** The differences of clinical pathological characteristics between LRPS-high and LRPS-low.

Variables	Patients (*n*)	LRPS-high (*n*)	LRPS-low (*n*)	*p*-value
Age at diagnosis (years)	498	248	250	>0.9999
≤60	243	121	122	
>60	255	127	128	
Gender	498	248	250	0.6851
Male	366	180	186	
Female	132	68	64	
Alcohol history	487	241	246	0.0657
Yes	330	173	157	
No	157	68	89	
Smoking history	493	244	249	>0.9999
Yes	488	242	246	
No	5	2	3	
Clinical T stage	483	243	240	0.3943
T1–T2	175	93	82	
T3–T4	308	150	158	
Clinical stage	484	243	241	0.3907
I–II	113	61	52	
III–IV	371	182	189	
Histological stage	479	241	238	0.9163
G1–G2	358	181	177	
G3–G4	121	60	61	
Lymphatic metastasis	405	212	193	0.6143
With	235	126	109	
Without	170	86	84	
Distant metastasis	473	238	235	>0.9999
With	5	3	2	
Without	468	235	233	
Lymphovascular invasion	337	181	156	0.6492
With	119	66	53	
Without	218	115	103	
Perineural invasion	349	185	164	0.0027**
With	164	101	63	
Without	185	84	101	
Recurrence	103	55	48	0.0024**
With	40	29	11	
Without	63	26	37	

**, level of significance, p < 0.01.

**Figure 6:**
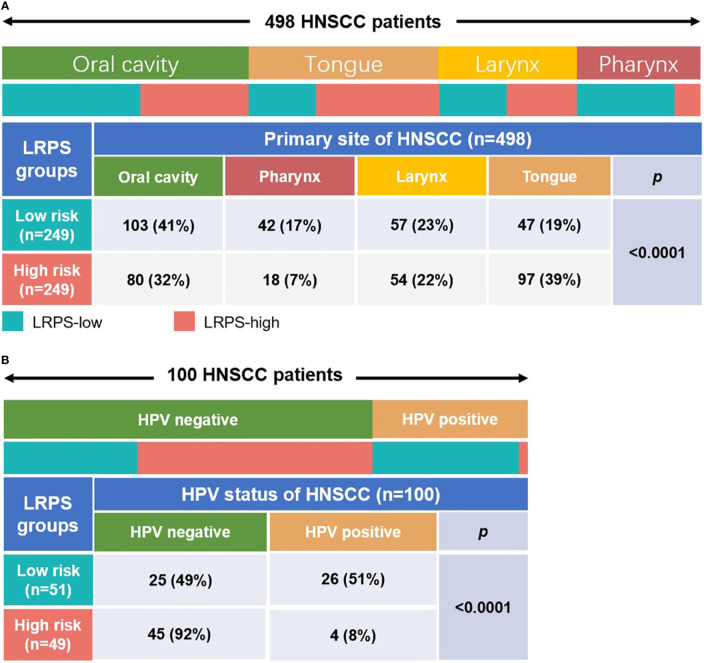
Distribution of primary tumor sites and the HPV status in different LRPS subgroups. **(A)** Heatmap and table showing the distribution of HNSCC primary sites (oral cavity, pharynx, larynx, and tongue) between the LRPS subgroups. **(B)** Heatmap and table showing the distribution of different HPV status between the two LRPS subgroups. The distributions of the primary site subtypes and HPV status in the LRPS subgroups were compared through the Fisher test.

Furthermore, based on the primary tumor sites, the data were classified into four subgroups: oral cavity, tongue, larynx, and pharynx. The results showed that the proportion of oral cavity and larynx samples were almost equally distributed between the high risk and low risk, but there were more cases of tongue and fewer pharynx in the high-risk than in the low-risk group (*p* < 0.0001, Fisher test; **Figure 6A**). Meanwhile, the samples with HPV test results were subdivided into positive and negative groups, and the data were performed to analyze the association between HPV status and the lipid signature. Surprisingly, we found that almost all HPV-positive samples showed a low risk for LRPS, while HPV-negative samples had a high risk for LRPS (*p* < 0.0001, Fisher test; **Figure 6B**).

### Molecular and Immune Characteristics in Different LRPS Subgroups

**Figure 7:**
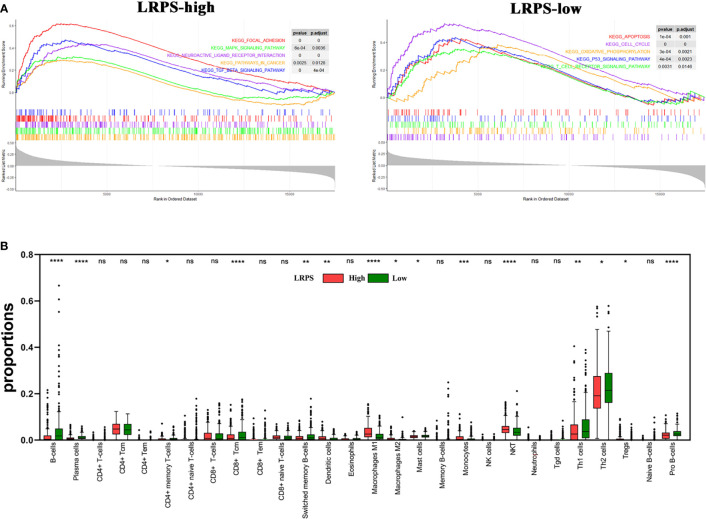
Molecular characteristics of LPRS subgroups. **(A)** Gene sets enrichment in the LRPS-high and LRPS-low groups, respectively (*p* < 0.05, FDR < 0.25). **(B)** The proportions of immunocytes within tumor microenvironment in different LRPS subgroup (ns, no significance; *p < 0.05; **p < 0.01; ***p < 0.001; ****p < 0.0001). NK cells, natural killer cells; NKT, natural killer T cells; Tgd cells, gamma delta T cells.

Since the lipid signature could increase the risk for recurrence, we sought to illuminate potential mechanisms regulating cancer relapse. Different LRPS groups were performed to GSEA and xCell analyses. We observed that the LRPS-high group significantly positively correlated to focal adhesion, MAPK signaling pathway, neuroactive ligand–receptor interaction, cancer-related pathway, and TGFβ signaling pathway. The LRPS-low group was mainly enriched and negatively related to apoptosis, cell cycle, oxidative phosphorylation, p53 signaling pathway, and T-cell receptor signaling pathway (**Figure 7A**, *p* < 0.05, FDR < 0.25).

xCell analyses revealed that compared with adjacent normal tissues, the tumors that had a high LRPS score were more infiltrated in NKT, dendritic cells, monocytes, Treg, and M1 and M2 macrophages, which is in line with the inflammatory niche of the HNSCC tumor microenvironment. In addition, a proportion of B cells and CD4^+^ T effector cells including Th1 and Th2 significantly decreased in the LRPS-high group compared with the LRPS-low group, implicating that there is a suppressive immunity in the LRPS-high group (**Figure 7B**). Notably, hematopoietic stem cells (HSCs) and mesenchymal stem cells (MSCs) were observed enriched in the LRPS-high group (*p* < 0.001). The above results indicated a possible changed immune milieu of primary tumor sites with an increased risk for HNSCC progression.

**Figure 8:**
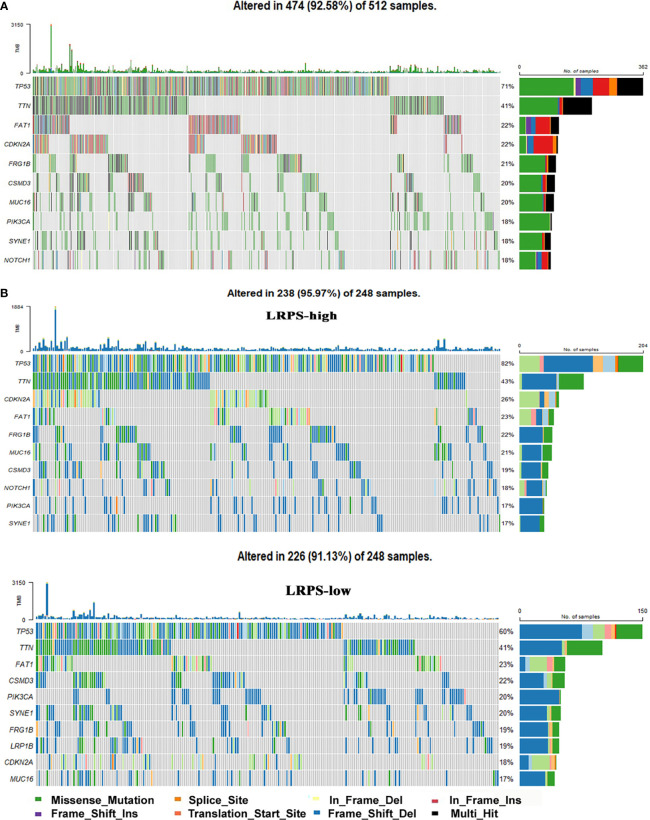
Genomic mutations in the LRPS. **(A)** Genomic mutation signature in the patients with HNSCC from the TCGA database. **(B)** Significantly mutated genes in LRPS-high and LRPS-low subgroups. The top 10 mutated genes are listed; the right shows mutation percentage and the top shows the overall mutation rates of different cohorts.

Finally, gene mutations were further analyzed to explore the molecular nature of the LRPS subgroups, and the top 10 genes with the highest mutation rates were identified (**Figure 8A**). Genomic analysis showed that HNSCC endowed a high frequency of TP53 mutations, as high as about 71%, suggesting a vital role on tumor bioactivities. Our results showed a higher mutation rate of TP53 in LRPS-high patients than those with low LRPS score (82% *vs.* 60%), underlying a potential crosstalk between altered lipid metabolism and TP53 status. Missense variations were the most popular in both LRPS-high and -low groups. Importantly, TP53 showed a significantly higher mutation rate in the LRPS-high than in the LRPS-low group (**Figure 8B**, *p* < 0.0001, Fisher’s exact test). In addition, TTN, CDKN2A, FAT1, FRGB1, MUC16, CSMD3, PIK3CA, and SYNE1 were higher than 16% in both groups. The correlation between LRPS score and total mutation burden (TMB) was further explored, suggesting that the LRPS score was slightly correlated with total mutation burden (*r* = −0.11, *p* = 0.015, **Figure S4**).

## Discussion

The first new finding of the manuscript is that we identify the novel lipid prognostic signature of ADCY2, LIPE, and OLR1, which can predict the survival and prognosis of HNSCC patients as an independent effective prognostic factor. Meanwhile, our data may explain how lipidomics affects the prognosis and survival of patients with HNSCC through affecting tumor microenvironment *via* immunosuppression.

In recent years, lipid metabolism has come into a sharp focus on cancer initiation and progression owing to its essential role in HNSCC and striking contribution to cancer development. In this study, we, for the first time, identified a novel LRPS of HNSCC through univariate and multivariate Cox analyses, which were performed to analyze the lipid-related DEGs as predictors for survival in TCGA patients with HNSCC. ADCY2, OLR1, and LIPE significantly predicted the overall survival of HNSCC among the lipid DEGs. When the three genes were combined to indicate the prognosis of HNSCC patients, it showed that the LRPS-high group was highly related to poor prognosis.

OLR1 is a stimulator of epithelial–mesenchymal transition (EMT) and involved in PPAR pathway, regulated by the secondary messenger cyclin adenosine monophosphate (cAMP). OLR1 promotes migration and metastatic spread in different pathways, such as TBC1D3/OLR1/TNFα/NF-κB, OLR1/c-Myc/HMGA2, oxLDL/OLR1/VEGF-C, and PI3K/Akt/GSK3β (32–36). Recently, LOX-1Δ4, an alternative OLR1 isoform, has been shown to directly drive non-tumorigenic breast epithelial cells into fast proliferation status (37). More importantly, OLR1 is also reported to be positively correlated with the occurrence of lymphatic metastases in pancreatic cancers (38).

Adenylate cyclase 2 (ADCY2) encodes the adenylate cyclase that catalyzes ATP to transform to the second messenger cyclic adenosine monophosphate (cAMP). The latter is a crucial signal in cell fate, inflammation, and many other bioactivities, and is also greatly involved in the growth and differentiation of MSCs. Zhao et al. have reported E2-induced ADCY2 as a positive regulator in MSCs (39). In colorectal cancer, ADCY2 could also be an important prognostic marker (40).

Lipase E, hormone-sensitive type (LIPE) increases both the levels of free cholesterol and free fatty acids, and plays an important role in adipocyte function and lipid and glucose homeostasis (41). More importantly, LIPE encodes the rate-limiting enzyme of lipolysis, and homozygous null mutation of LIPE results in marked inhibition of lipolysis, leading to multiple symmetric lipomatosis (42). Our studies showed that LIPE played a central role in the protein–protein interactions of the DEGs, significantly related to the survival rate of patients with HNSCC.

The second new finding of the study is that the LRPS can indicate the type of the infiltrated immune cells in the HNSCC tumor microenvironment. Comprehensive analyses indicated a diverse characteristic of LRPS subgroups. Lumps of the LRPS-high group showed a higher infiltration of inflammation-associated cells, including dendritic cells, M1 and M2 macrophages, and monocytes, yet a lower proportion of immunocytes (B cells and pro B cells, CD8+ Tcm) compared with the LRPS-low group. The finding is also supported by the new concept that the infectious, chronic irritated, and inflammatory infiltration induces cancer and promotes neoplastic risk.

Macrophages are the main source of tissue repairment-related growth factors and cytokines after activation, such as TGFβ1, TNFα, TGFα, and IL1 (43). These factors partly contributed to carcinogenesis *via* different signaling pathways. Numerous studies further showed a strong correlation between macrophage abundance and poor cancer prognosis, including thyroid cancer, lung cancer, and hepatocellular cancer (44–47). Compromised immunity was also observed in our results, consistent with the research that shows that high-fat diet-induced obesity accelerates tumor growth by impairing CD8+ T-cell function (48). These observations could partly elucidate that the patients with LRPS high score were more subject to a poor survival because of the inflammatory-rich and immunodeficient conditions.

Intriguingly, we found that LRPS-low harbors more Th1 and Th2 cells and fewer Treg cells in HNSCC. By contrast, LRPS-high has more Treg cells, consistent with the results from Whiteside group, which found a large number of Treg cells in the peripheral circulation of patients with HNSCC (49). Treg cells serve as one of the culprits that suppress anti-tumor immune response. Tumor within a niche of Treg cells is recognized as an unfavorable factor of cancer prognosis (50).

We further found that the patients with high LRPS score were more susceptible to recurrence because of increased infiltration of MSCs and HSCs in the tumor microenvironment. Recent data have proposed lipid metabolic rewiring as a new hallmark of cancer stem cells (CSCs) owing to its modification on stem-like cells’ properties (51).

Taken together, these results affirmed that abnormal lipid metabolism exerts a great impact on immune cells’ function in the tumor microenvironment, just influencing the progression and prognosis of HNSCC.

The third new finding of the study is that LPPS score can interpret the TP53 status of HNSCC. Our results showed that there were fewer LRPS-high samples in HPV-positive HNSCCs, which was in accordance with the negative relationship between HPV status and p53 mutation frequency (52). We also found a significant higher mutation rate of TP53 in LRPS-high patients than those with low LRPS score, underlying a potential crosstalk between altered lipid metabolism and TP53 status. Wild-type p53 supervises the cell damage response to various stimuli, and recent findings increasingly link p53 to lipid metabolism. P53 suppresses lipid biosynthesis *via* inhibiting lipogenesis, yet induces fatty acid oxidation as an alternative energy source to glycolysis in the condition of nutritional deficiency (53, 54), implicating p53 as a positive regulator of catabolism (increase fatty acid levels) and an inhibitor of anabolism (decrease fatty acid levels) in the process of fatty acid metabolism. Otherwise, loss of p53 can lead to cell malignant transformation. As expected, mutated p53 exert a great impact on carcinogenesis through regulating gene transcription related to cell cycle, DNA repair, immunity and energetic activities, and so on. This gain of function of mutated p53 has been validated in various human cancers including breast, prostate, colon, pancreas, and head and neck cancers (55–60). Our data further supported that p53 mutations did cooperate with abnormal lipid metabolism to promote cancer progression in HNSCC, though more laboratory investigations are needed in the future.

Though the LRPS has great potential for predicting HNSCC survival and p53 status, there are some limitations. The training and validation cohorts were retrospective, and more findings need to be validated prospectively. Moreover, the value of LRPS is not validated by *in vitro* and *in vivo* assays. Therefore, more studies are needed in the future.

## Conclusion

Our data confirmed that the three lipid-related genes play a pivotal role in tumorigenesis and recurrence of HNSCC, potentially by suppressing anti-tumor immunity and reflecting TP53 mutations status. LRPS has a potential to be a promising indicator of overall survival, prognosis, TP53 status, and immune characteristics in HNSCC, and perhaps could monitor and guide the treatment efficacy and prognosis of HNSCC in the future.

## Data Availability Statement

Publicly available datasets were analyzed in this study. These data can be found here:. The original datasets comparing the mRNA expression profiles between tumors and adjacent normal tissues were obtained from the GEO databases (GSE30784, GSE37991 and GSE65858) and the TCGA dataset. The microarray data of GSE30784 and GSE37991 were based on GPL570 (Affymetrix Human Genome U133 Plus 2.0 Array) and GPL6883 (Illumina HumanRef-8 v3.0 expression beadchip) and GPL10558 (Illumina HumanHT-12 V4.0 expression beadchip), respectively. The corresponding clinical information of patients with HNSCC was also acquired from TCGA database (up to July 19, 2019). A total of 498 HNSCC patients with detailed follow-up time were included for the following analyses.

## Author Contributions

Conceptualization: YW, ZM, and SZ Data curation: XG. Formal analysis: XG, NZ, CD, LD, XZ, and YZ. Supervision: YW. Writing—original draft: XG. Writing—review and editing: YW, ZM, and SZ. All authors contributed to the article and approved the submitted version.

## Funding

This research was funded by Research Grants from the Natural Science Foundation of Beijing Municipality (grant No. 7182181), the National Nature Science Foundation of China (grant Nos. 81772873, 81970920, and 81900983), and Shanghai Science and Technology Young Talents Sailing Program (19YF1442500).

## Conflict of Interest

The authors declare that the research was conducted in the absence of any commercial or financial relationships that could be construed as a potential conflict of interest.

## Publisher’s Note

All claims expressed in this article are solely those of the authors and do not necessarily represent those of their affiliated organizations, or those of the publisher, the editors and the reviewers. Any product that may be evaluated in this article, or claim that may be made by its manufacturer, is not guaranteed or endorsed by the publisher.

## Supplementary Material

Supplementary Figure 1 Lipid-related differentially expressed genes (DEGs) in HNSCC of GEO datasets. **(A, B)** showed 65 lipid DEGs in GEO database, GSE30784 and GSE37991 respectively (p<0.01, |logFC|>2). The color from blue to red represented the gene expressions from high to low between tumors *vs.* normal tissues.

Supplementary Figure 2 LRPS in the GEO dataset. **(A)** Survival analysis showed a significant difference between high-risk group and low-risk group in GSE65858 dataset (p=4.304×10-5). **(B)** The 5-year ROC value in the GEO group is 0.811.

Supplementary Figure 3 LRPS in the TCGA validation dataset. **(A, B)** Three mRNA expression and risk score division in the TCGA validation dataset. **(C)**. Survival analysis showed a significant difference between high-risk group and low-risk group in TCGA validation dataset (p=1.337×10-5). **(D)**. The 3-year, 5-year and 10-year ROC values in the validation group are 0.625, 0.618 and 0.592, respectively.

Supplementary Figure 4 The relationship between LRPS and tumor mutational burden. Correlation analysis between LRPS score and total tumor mutational burden (TMB) in HNSCC from TCGA database.

File S1 List of full lipid-related pathways and the corresponding genes from KEGG database.

File S2 List of protein–protein interaction (PPI) results from 65 lipid DEGs and two key modules within the PPI.

File S3 The LRPS expression and the corresponding clinical information of high-risk and low-risk groups in the model dataset and the validation dataset, respectively.

File S4 Results of all DEGs in HNSCC using univariate Cox analysis.

File S5 Kaplan–Meier results of LRPS-high and -low subgroups in the model dataset and validation dataset, respectively.

Supplementary Table 1 Clinical pathological characteristics comparison between the testing dataset and validation dataset.

## Abbreviations

HNSCC, Head and neck squamous cell carcinoma; TCGA, The Cancer Genome Atlas; GEO, Gene expression Omnibus; LRPS, Lipid-related prognostic signature; NKT, Natural killer T cell; DEGs, Differentially expressed genes; GSEA, Gene-set enrichment analysis; GO, Gene ontology; DAVID, The Database for Annotation, Visualization, and Integration Discovery; BP, Biological processes; CC, Cellular component; MF, Molecular function; PPI, Protein–protein interaction; HSC, Hematopoietic stem cells; MSC, Mesenchymal stem cells; TMB, Total mutation burden; EMT, Epithelial–mesenchymal transition; ADCY2, Adenylate cyclase 2; LIPE, Lipase E, hormone sensitive type; CSCs, Cancer stem cells.
