# Supplementary figures and images for: A Novel Lipid Prognostic Signature of ADCY2, LIPE, and OLR1 in Head and Neck Squamous Cell Carcinoma
Source: Front Oncol. 2021 Nov 25;11:735993. doi: 10.3389/fonc.2021.735993 (PMC8655234; doi:10.3389/fonc.2021.735993)

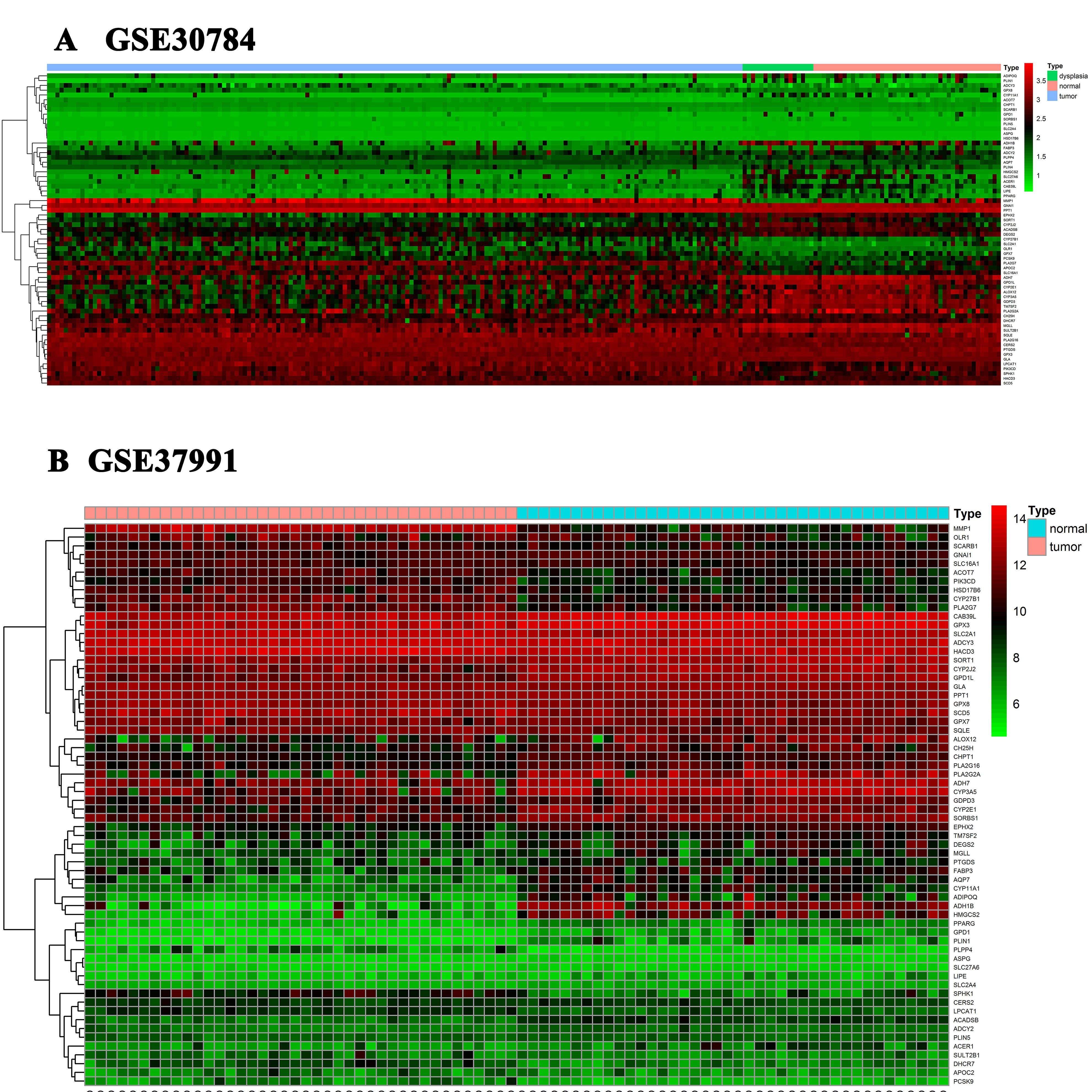

Supplement: Supplementary Figure 1 — Lipid-related differentially expressed genes (DEGs) in HNSCC of GEO datasets. (A, B) showed 65 lipid DEGs in GEO database, GSE30784 and GSE37991 respectively (p<0.01, |logFC|>2). The color from blue to red represented the gene expressions from high to low between tumors vs. normal tissues. [file Image_1.jpeg]

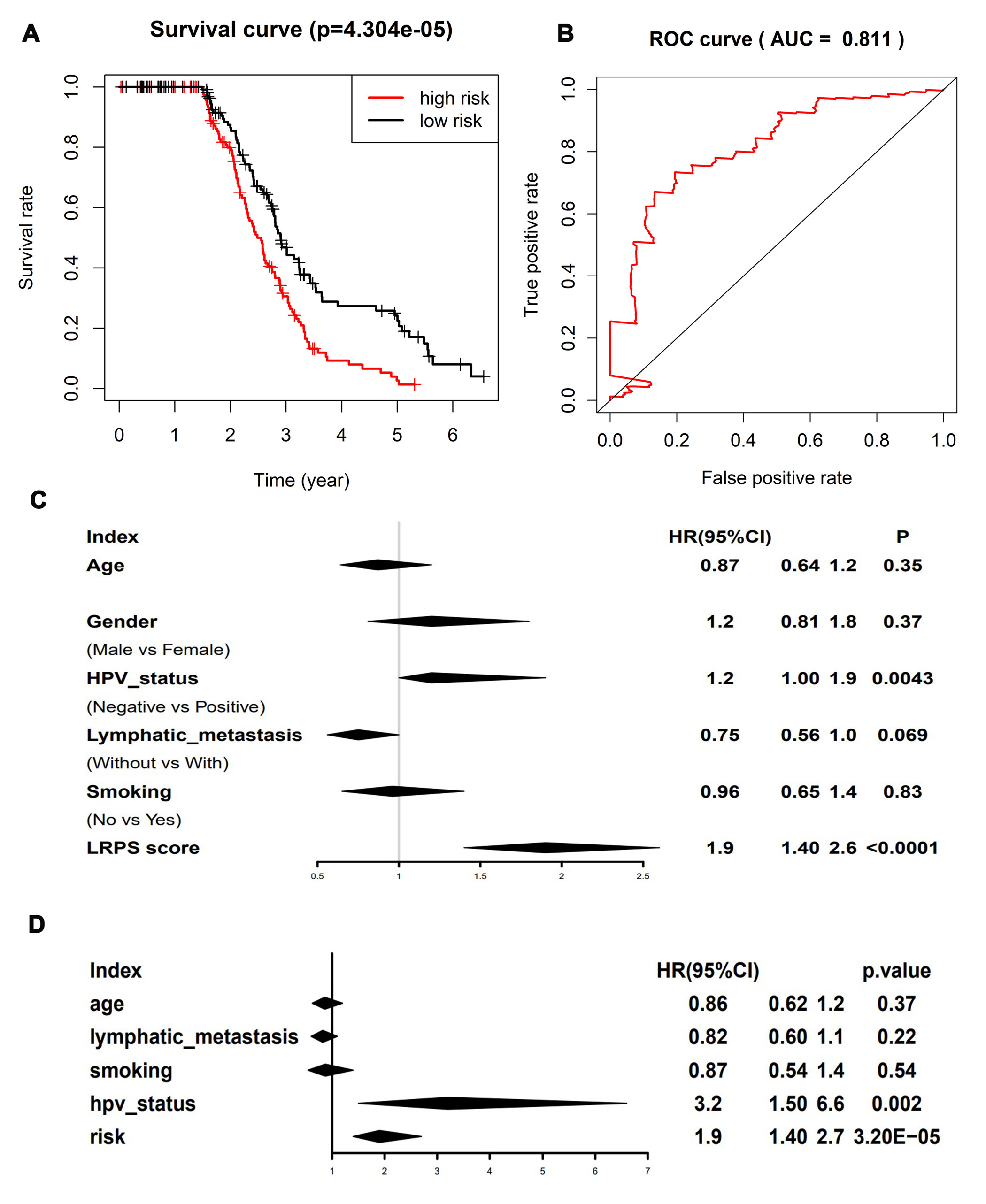

Supplement: Supplementary Figure 2 — LRPS in the GEO dataset. (A) Survival analysis showed a significant difference between high-risk group and low-risk group in GSE65858 dataset (p=4.304×10^-5^). (B) The 5-year ROC value in the GEO group is 0.811. [file Image_2.jpeg]

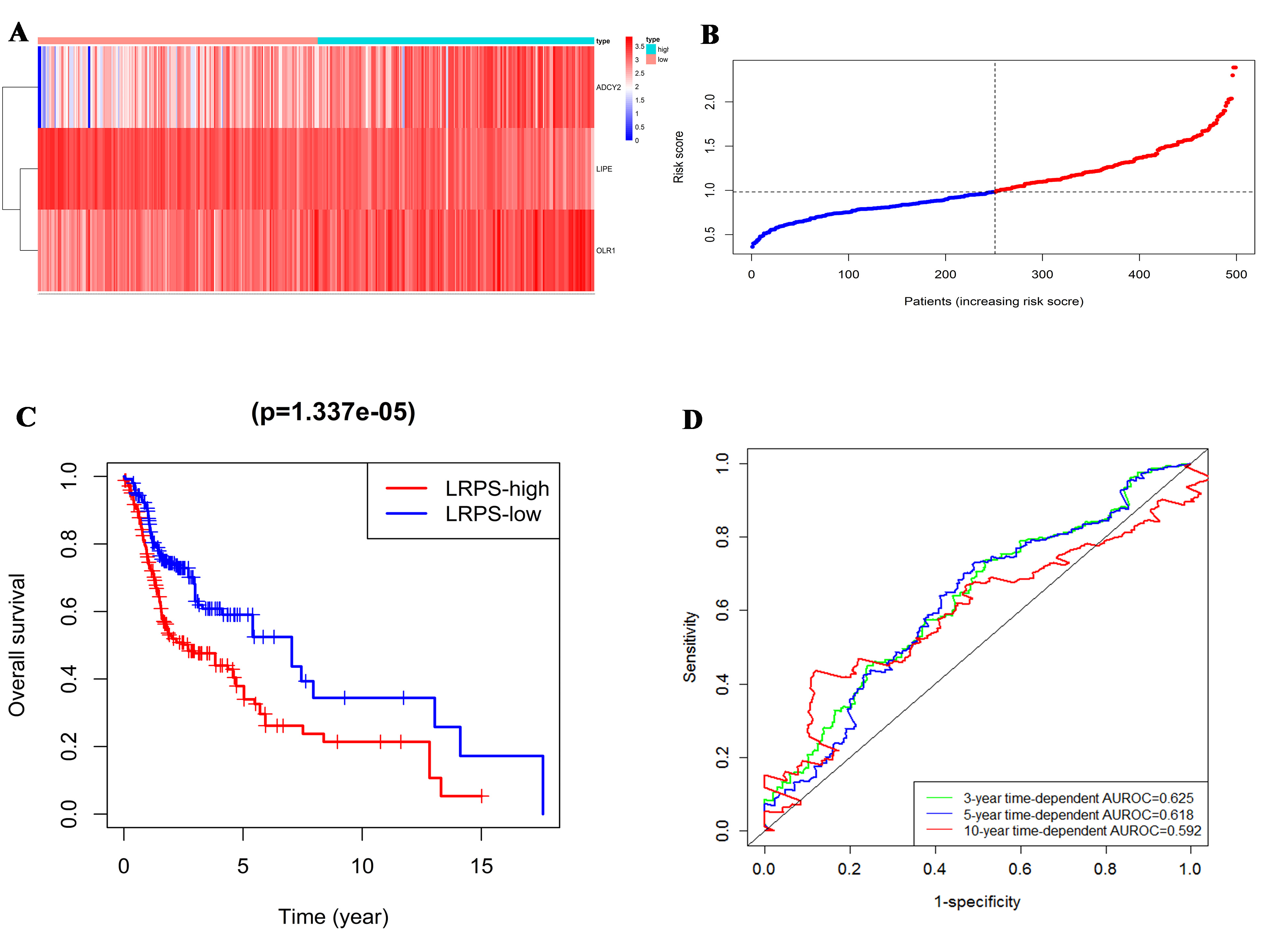

Supplement: Supplementary Figure 3 — LRPS in the TCGA validation dataset. (A, B) Three mRNA expression and risk score division in the TCGA validation dataset. (C). Survival analysis showed a significant difference between high-risk group and low-risk group in TCGA validation dataset (p=1.337×10^-5^). (D). The 3-year, 5-year and 10-year ROC values in the validation group are 0.625, 0.618 and 0.592, respectively. [file Image_3.jpeg]

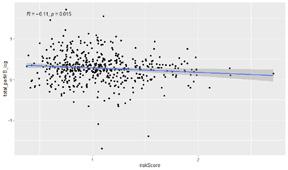

Supplement: Supplementary Figure 4 — The relationship between LRPS and tumor mutational burden. Correlation analysis between LRPS score and total tumor mutational burden (TMB) in HNSCC from TCGA database. [file Image_4.jpeg]
