# Supplementary material for: A Novel Lipid Prognostic Signature of ADCY2, LIPE, and OLR1 in Head and Neck Squamous Cell Carcinoma
Source: Front Oncol. 2021 Nov 25;11:735993. doi: 10.3389/fonc.2021.735993 (PMC8655234; doi:10.3389/fonc.2021.735993)
Supplement: Supplementary Table 1 — Clinical pathological characteristics comparison between the testing dataset and validation dataset. [file Table_1.docx]

|  | Testing group(n=498) | Validation group(n=498) | *P*-value |
| --- | --- | --- | --- |
| Death(n,%) | 281(56.43) | 282(56.62) | 0.976 |
| Survival time(mean±SD,years) | 2.50±2.42 | 2.46±2.48 | 0.985 |
| Age(mean±SD,years) | 61.12±11.87 | 61.08±11.09 | 0.932 |
| Gender(male/female) | 366/132 | 356/142 | 0.999 |
| Clinical stage(NA/I/II/III/IV) | 14/19/94/101/270 | 12/23/94/104/265 | 0.965 |
| PT(NA/T_0_/T­_1_/T_2_/T_3_/T_4_) | 22/34/45/131/95/171 | 18/38/44/128/101/169 | 0.869 |
| PN(NA/NX/NO/N1/N2/N3) | 24/69/170/65/163/7 | 19/69/189/64/153/4 | 0.890 |
| PM(NA/MX/M0/M1) | 251/61/185/1 | 262/51/183/2 | 0.941 |

**Table S1 Clinical pathological characteristics comparison between testing dataset and validation** **dataset**

PT, pathological T stage; PN, pathological N stage; PM, pathological M stage.
